# Indices of neighborhood disadvantage and individual cancer control behaviors among African American adults

**DOI:** 10.1093/jncics/pkaf015

**Published:** 2025-02-28

**Authors:** Bernard F Fuemmeler, Joseph Boyle, Carrie A Miller, Debarchana Ghosh, Cheryl L Knott

**Affiliations:** Department of Family Medicine and Population Health, Virginia Commonwealth University, Richmond, VA 23219, United States; Massey Comprehensive Cancer Center, Richmond, VA 23219, United States; Department of Family Medicine and Population Health, Virginia Commonwealth University, Richmond, VA 23219, United States; Massey Comprehensive Cancer Center, Richmond, VA 23219, United States; Department of Family Medicine and Population Health, Virginia Commonwealth University, Richmond, VA 23219, United States; Massey Comprehensive Cancer Center, Richmond, VA 23219, United States; Department of Geography, University of Connecticut, Storrs, CT 06269, United States; Department of Behavioral and Community Health, University of Maryland, College Park, College Park, MD 20742, United States

## Abstract

**Background:**

Emerging literature notes the importance of neighborhood-level factors for cancer control behaviors beyond that of individual factors. Markers of neighborhood-level disadvantage have been linked to greater likelihood of nonsalutary cancer control behaviors. There has been less examination of many neighborhood factors simultaneously, which more accurately reflects individuals’ daily experiences. We estimated associations of neighborhood deprivation indices with cancer control behaviors, identifying the relative importance of neighborhood-level deprivation index components for these outcomes.

**Methods:**

We used data from the Religion and Health in African Americans study, a national probability sample of African American adults. We separately considered 4 screening and 4 prevention behaviors as outcomes. We constructed neighborhood deprivation indices using census tract-level data and estimated their associations with outcomes using bayesian index models, adjusting for individual-level covariates. We reported odds ratios (ORs), credible intervals, and exceedance probabilities.

**Results:**

Participants in our sample engaged in relatively high levels of screening behaviors and lower levels of prevention behaviors. Neighborhood deprivation indices were statistically significantly associated with a greater likelihood of binge drinking (OR = 1.13, exceedance probability = 98.5%), smoking (OR = 1.07, exceedance probability = 99.4%), and insufficient colonoscopy (exceedance probability = 99.9%), Papanicolaou (exceedance probability = 99.7%), and prostate-specific antigen (exceedance probability = 99.1%) screening. Within neighborhood deprivation indices, median household income, percentage of individuals without some college education, and percentage of individuals unemployed received large estimated importance weights.

**Conclusion:**

We identified statistically significant associations between neighborhood disadvantage and nonsalutary cancer control behaviors as well as important neighborhood-level deprivation index components for each outcome. These and similar findings from future studies should be used to target specific neighborhood factors for specific cancer control behaviors rather than using a one-size-fits-all approach.

## Introduction

Key modifiable risk factors and cancer prevention practices, such as screening, contribute to more than 50% of cancer incidence in the United States.^1^^,^^2^ Disparities in cancer control behaviors lead to inequities in cancer outcomes, with lower rates of screening and preventive measures in low-income and racially or ethnically minoritized populations contributing to higher incidence and mortality.^3-6^ Lifestyle factors such as smoking, physical inactivity, and poor diet are more prevalent in socioeconomically disadvantaged neighborhoods, further exacerbating cancer risks. Social, systemic, and structural neighborhood characteristics often drive cancer outcomes.^7-10^ Understanding the drivers that can be modified is essential for improving cancer control behaviors among minoritized populations and informing strategies to reduce disparities.

Understanding the social drivers of cancer control behaviors requires multilevel analysis, considering both contextual factors and individual differences. Interest in examining neighborhood and other area-level factors related to cancer outcomes is growing.^3^^,^^4^^,^^7-9^^,^^11-13^ Emerging studies highlight that neighborhood deprivation, segregation, and other socioeconomic factors captured by area-level variables influence cancer outcomes. Rigorous analytical methods accounting for multiple area-level variables are needed to corroborate and expand these initial findings. Such approaches can enhance precision population health by identifying key social drivers of cancer control behaviors and targeting them for community-based interventions.

In a recent study, Knott et al. examined individual and area-level variables related to cancer control behaviors in the Religion and Health in African Americans (RHIAA) cohort, finding that neighborhood racial and ethnic diversity, median income, and home ownership strongly contributed to these outcomes beyond individual factors.^14^ Particularly, separate indicators of neighborhood disadvantage were strongly associated with a higher likelihood of smoking, while men in wealthier neighborhoods with older residents and greater home ownership were much more likely to report prostate-specific antigen (PSA) testing.

Decoupling the effects of various neighborhood variables on cancer control behaviors and outcomes is challenging because these factors tend to be correlated. Recent methodologies can handle many correlated exposures simultaneously, relevant for neighborhood socioeconomic indicators.^15-17^ This paper aims to simultaneously consider the impact of neighborhood deprivation with its constituent area-level components on cancer control behaviors in African Americans among RHIAA cohort participants using bayesian index models.^8^^,^^13^^,^^18^^,^^19^ These models will quantify the neighborhood-level effects of deprivation on cancer control behaviors while simultaneously accounting for individual-level covariates.

## Methods

### Participant data

Data came from the RHIAA study.^20^ Supported by the National Cancer Institute (NCI), the RHIAA study was originally designed to evaluate the role of psychosocial resources, such as religious involvement, in predicting cancer control behaviors in African Americans. Though not designed to be a representative sample in the pure sense, probability-based sampling was employed to recruit African American adult participants from all US states. The sampling frame was established from publicly available data sources, and participants were contacted by telephone and screened for eligibility. Enrollment occurred between 2008 and 2010. The resulting study sample was more heavily weighted to participants living in the southern United States, which is consistent with US Census Bureau data on geographic dispersion of African American households.^21^ In addition, the RHIAA sample comprised more women than men, and the sample educational level was comparable to national data.^22^ Further details of sampling and recruitment methods are provided elsewhere.^23^^,^^24^ Professional interviewers obtained informed verbal consent from all participants. Our sample consisted of 3117 participants who spoke English, self-identified as African American, were 21 years of age or older, had no history of cancer, and had provided their residential address. In the present analysis, we excluded participants from 3 US Census Bureau divisions (New England, Pacific, and Mountain) due to small numbers of participants from these regions. Survey interviews lasted approximately 1 hour and included questions about cancer risk, prevention, and screening behaviors. The University of Maryland Institutional Review Board approved both RHIAA and the present study. All procedures followed were in accordance with the ethical standards of the Declaration of Helsinki.

### Outcome measures

We evaluated 4 cancer prevention outcomes, including whether participants had had at least 3 heavy drinking days in the past month and whether participants had smoked at least 100 cigarettes in their lifetime.^25^^,^^26^ These items, taken from the Behavioral Risk Factor Surveillance System,^27^ were shown to have adequate test-retest reliability among African American participants.^28^ We also evaluated whether participants consumed at least 5 fruits and vegetables per day using an adapted version of the NCI 5-A-Day Survey^29^^,^^30^ and whether participants completed at least 150 minutes of moderate to vigorous physical activity per week. The International Physical Activity Questionnaire^31^^,^^32^ was used to determine physical activity. For the screening outcomes, ever having had a colonoscopy, mammogram, Papanicolaou test, and PSA test were collected. Screening questions were assessed according to relevant sex and age guidelines at the time of survey administration. See Supplementary Methods for additional details on outcomes.

### Neighborhood-level exposures

To measure exposure to multidimensional disadvantages and risk factors, we built a custom neighborhood-level deprivation index using nationally representative and publicly available data. We used participant residential addresses to obtain spatial coordinates, then used these coordinates to determine the census tract that contained participants’ address. Further details about this procedure are provided elsewhere.^14^ For this study, we defined the census tract as constituting a participant’s neighborhood. See Supplementary Methods for additional details on census tracts.

Neighborhood variables were obtained from the American Community Survey (ACS), using 5-year estimates ending in 2010. We used variables similar to those in other neighborhood indices,^8^^,^^13^ such as the Area Deprivation Index (ADI)^33^ and Social Vulnerability Index^34^ (see Supplementary Material for details). The 12 neighborhood variables in our neighborhood-level deprivation index were median household income, percentage of households with public assistance income, percentage of Black residents, percentage of renter-occupied housing units, percentage of residents without some college education, percentage of residents unemployed, percentage of single-parent households with children under 18 years of age, median house value, percentage of households with an income to poverty ratio below 1% crowded households, percentage of vacant housing units, and median rent as a percentage of income. We included the percentage of Black residents to represent concentrations of social and economic disadvantage resulting from anti-Black racism.^35-38^ We inverted the income and house value variables by subtracting them from their sample maximum to ensure that all components were in the direction of larger values reflecting greater disadvantage.

### Statistical analyses

Data were summarized with descriptive statistics, using medians (IQRs) for continuous variables, with frequency and percentage for categorical variables. Then, we calculated the sample prevalence of each cancer control behavior and produced a correlation matrix for the neighborhood-level disadvantage index components.

Then, we estimated the associations between neighborhood disadvantage and cancer control outcomes using validated bayesian index models.^16^ These models provide better goodness of fit and accuracy for correlated variables, such as neighborhood and socioeconomic variables.^39^^,^^40^ Bayesian index models estimate both the association between an index and the outcome as well as the relative importance of each component in the index, enabling comparison of the importance of each index component with the outcome. The importance weights were defined to be between 0 and 1 and sum to 1 within each index, providing intuitive interpretations of variable contribution to the index. See Supplementary Methods for details on bayesian index models. We adjusted for participants’ covariates, specifically adjusting for (self-reported) gender (in models for outcomes relevant to men and women), age (mean centered), and education (reference: college graduate).

We calculated posterior means and 95% credible intervals as well as the exceedance probability for these parameters, which estimates the posterior probability that a parameter exceeds the null value.^41^ In addition, we calculated the predicted probabilities of each outcome based on deciles of the neighborhood-level deprivation index, holding model covariates at their mode/mean sample values. We estimated model parameters using Just Another Gibbs Sampler, with the R package *rjags*^42^ in R, version 4.3.1, software (R Foundation for Statistical Computing)^.43^ A 5% statistical significance level was used; tests of significance were 2 sided.

### Supplemental analyses

We conducted 5 supplemental analyses to validate our primary findings and explore additional hypotheses. First, we used a simplified neighborhood-level deprivation index with equal component weights, implying that the index was a simple average of its components, and compared the goodness of fit to the bayesian index model using deviance and the Deviance Information Criterion.^44^^,^^45^ Second, we fit bayesian index models with an interaction term between gender and neighborhood-level deprivation index to explore whether gender moderated the associations between neighborhood disadvantage and cancer control outcomes. Third, we controlled for individual-level employment and subsequently for individual-level income in all models to determine whether factors confounded associations identified in the primary analysis. Fourth, we used the ADI^33^ instead of the bayesian neighborhood-level deprivation index to explore the concordance between findings using the 2 indices. Finally, we repeated the main analysis using current smoking status as the smoking outcome variable.

## Results

### Participant characteristics

Table 1 describes characteristics of the sample. The median age of participants was 54 years. The majority of participants were female (62%) and had attained at least a high school diploma (87%). One-quarter were college graduates (25%). Most participants lived in relatively low-income (median household income = $23 400) census tracts with a high Black population (median percentage Black residents = 86%). Additional neighborhood characteristics for participants in the sample characterize a slightly high^46^ unemployment rate (median = 14%) as well as the prevalence of vacant housing units (median = 15%) and receipt of public assistance (median = 38%). The majority of participants lived in the South Atlantic (33.8%) and East North Central (20.9%) US Census regions.

**Table 1.** Summary of participant characteristics.

Variable	**Value** ^a^
Age, median (IQR), y	54 (45-64)
Gender, No. (%)	
Male	837 (37.7)
Female	1385 (62.3)
Education, No. (%)	
Middle school or less	60 (2.7)
Some high school	214 (9.6)
High school graduate	734 (33.0)
Some college	643 (28.9)
College graduate	560 (25.2)
Missing	11 (0.5)
Neighborhood variables, median (IQR)	
Household income^b^	23.4 (15.2-36.4)
Percentage with public assistance	38.1 (34.2-41.6)
Percentage of Black residents	86.4 (64.9-94.2)
Percentage of residents who are renters	37.9 (28.4-50.8)
Percentage of residents without some college	85.8 (82.4-89.1)
Percentage of residents who are unemployed	13.7 (9.4-19.4)
Percentage of single-parent households with children <18 y of age	16.6 (11.9-22.3)
House value^b^	92.5 (60.7-166.1)
Percentage of residents with a ratio of income to poverty <1	25.3 (14.7-36.5)
Percentage of crowded households	2.4 (0.8-4.8)
Percentage of vacant households	14.7 (9.3-21.5)
Rent as a percentage of income	35.2 (29.9-43.0)
Census region, No. (%)	
Middle Atlantic	385 (17.4)
East North Central	464 (20.9)
West North Central	69 (3.1)
South Atlantic	749 (33.8)
East South Central	252 (11.4)
West South Central	298 (13.4)
Prevention outcomes,^c^ No. (%)	
Alcohol binge (n = 2169)	132 (6.1)
Physical activity (n = 2123)	1420 (66.9)
Smoking (n = 2217)	979 (44.2)
Vegetable/fruit (n = 2222)	1307 (58.8)
Screening outcomes,^c^ No. (%)	
Colonoscopy (n = 1253)	408 (32.6)
Mammogram (n = 1149)	80 (7.0)
Papanicolaou test (n = 1355)	73 (5.4)
Prostate-specific antigen test (n = 596)	168 (28.2)

a Characteristics were tabulated using the sample for the outcome with the largest sample size (N = 2222).

b Units for median household income and median house value are thousands of US dollars.

c The outcome variables reflect the frequency of participants with a negative (eg, non–health-promoting) cancer control behavior.

Participants in our sample, particularly women, tended to have completed cancer screenings. Only 5% and 7% of age-eligible women had never completed a Papanicolaou test or mammogram, respectively. In addition, 28% of age-eligible men had not completed a PSA test, and 33% of the age-eligible sample had not completed a colonoscopy. Cancer preventive behaviors were less common, however. Although only 6% of participants reported at least 3 alcohol binge drinking days in the previous month, 67% reported insufficient moderate to vigorous physical activity, 59% reported insufficient fruit and vegetable consumption, and 44% had smoked at least 100 cigarettes in their lifetime.

The neighborhood-level deprivation index components were frequently correlated (see Figure S1). Of 66 pairwise correlations, 60 were statistically significant, 54 were positive, and 26 had an absolute magnitude of at least 0.25. The largest pairwise correlations were median household income, with the percentage of households having a ratio of income to poverty less than 1 (0.76) and a median house value (0.61).

### Prevention outcomes

**Figure 1.**
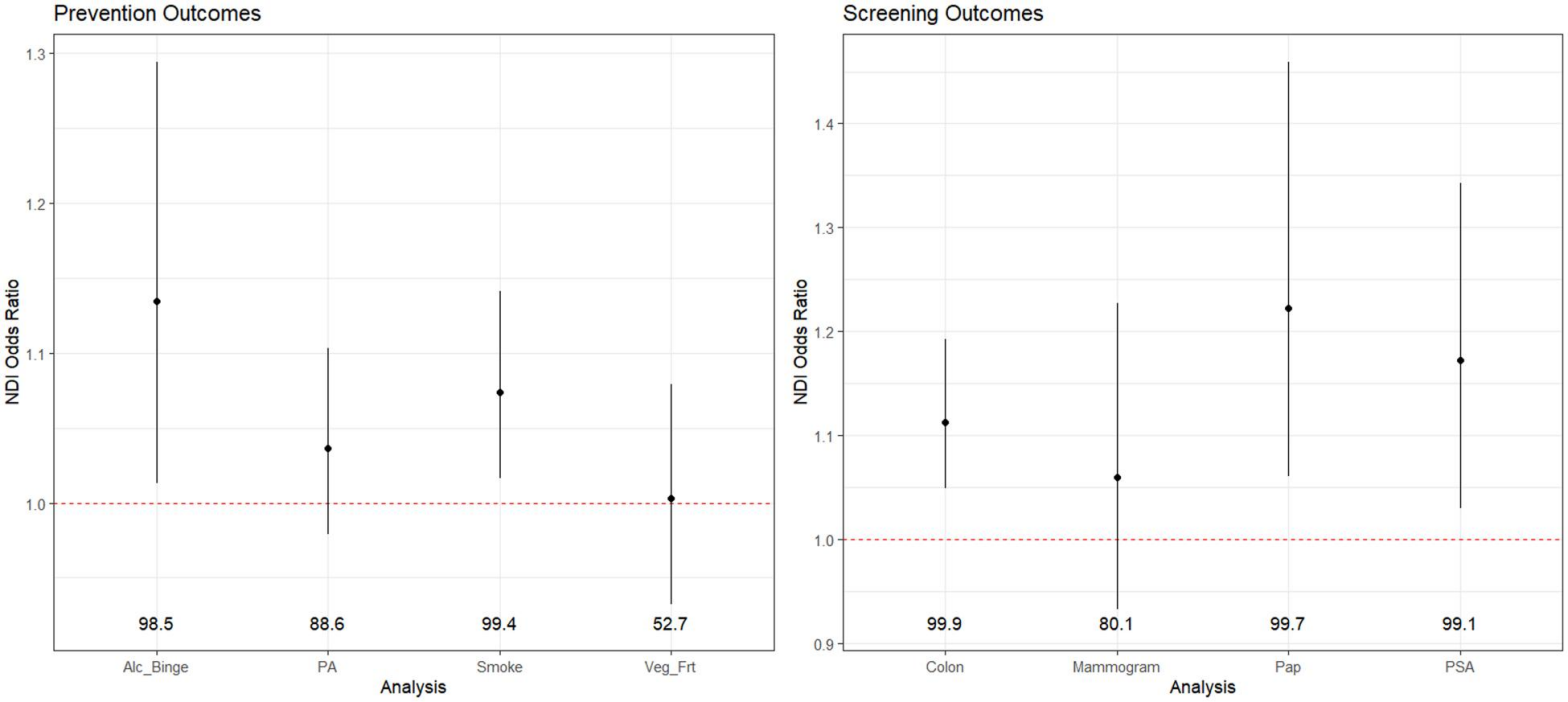
Summary of mean odds ratio and 95% credible interval for neighborhood disadvantage index, by cancer control outcome. The dashed line denotes null odds ratios. Numbers for each outcome denote the exceedance probability, which estimates the posterior probability that the quantity exceeds the null value. Abbreviations: NDI = neighborhood deprivation index; Alc_Binge = alcohol binge drinking; PA = physical activity; Veg_Frt = vegetables and fruits; PSA = prostate-specific antigen test.

**Figure 2.**
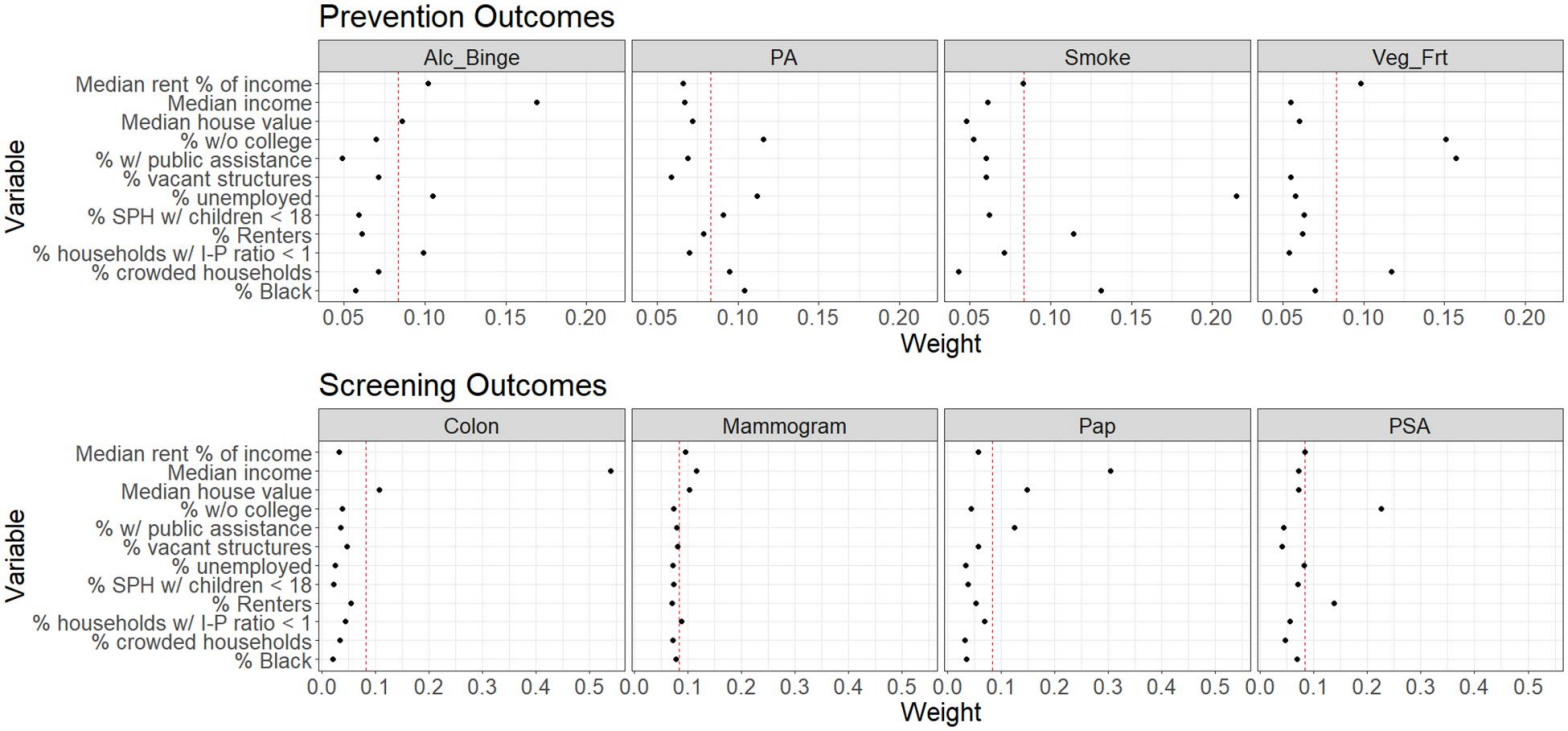
Summary of estimated importance weights in the neighborhood-level disadvantage index, by cancer control outcome. Modeled probabilities are of the nonsalutary outcome (eg, likelihood of reporting alcohol binge drinking; likelihood of reporting no mammogram) to be in the same direction as the modeled outcomes. Dashed line denotes the importance weight corresponding to the equal importance of all index components. Abbreviations: I-P = income to poverty; SPH = single-parent household. Alc_Binge = alcohol binge drinking; PA = physical activity; Veg_Frt = vegetables and fruits; PSA = prostate-specific antigen test.

The neighborhood-level deprivation index was statistically significantly associated with the likelihood of having had at least 3 alcohol binge drinking episodes in the past month (see Figure 1) (odds ratio [OR] = 1.13, 95% credible interval = 1.01-1.29, exceedance probability = 98.5%); within the index, median household income (0.169) received the majority of the weight (see Figure 2). In addition, the neighborhood-level deprivation index was statistically significantly associated with the likelihood of smoking at least 100 cigarettes (OR = 1.07, 95% credible interval = 1.02-1.14, exceedance probability = 99.4%); within the index, the percentage unemployed (0.215) and percentage Black residents (0.131) received the largest weights. Finally, the index was positively but not statistically significantly associated with the likelihood of insufficient physical activity (exceedance probability = 88.6%) and consuming insufficient fruits and vegetables (exceedance probability = 52.7%). The predicted probabilities of alcohol binge drinking and of smoking increased from 1.9% to 5.6% and from 30.2% to 44.7%, respectively, from the lowest to the highest deciles of the index (Figure S2).

### Screening outcomes

The neighborhood-level deprivation index was statistically significantly associated with the likelihood of not having had a colonoscopy (OR = 1.11, 95% credible interval = 1.05-1.19, exceedance probability = 99.9%); within the index, median household income (0.540) received the majority of the weight. In addition, the index was statistically significantly associated with the likelihood of not having had a Papanicolaou test (OR = 1.22, 95% credible interval = 1.06-1.46, exceedance probability = 99.7%); within the index, median household income (0.305) received the largest importance weight. The index was also statistically significantly associated with the likelihood of not having had a PSA test (OR = 1.17, 95% credible interval = 1.03-1.34, exceedance probability = 99.1%); within the index, the percentage of individuals without some college education (0.226) received the largest weight. Finally, the neighborhood-level deprivation index was positively but not statistically significantly associated with the likelihood of not having had a mammogram (exceedance probability = 80.1%). The predicted probabilities of colonoscopy, Papanicolaou test, and PSA test decreased from 77.8% to 57.3%, from 98.3% to 90.8%, and from 84.4% to 56.6% respectively, from the lowest to the highest deciles of the index.

### Supplemental analyses

Our first supplemental analysis justified the use of the bayesian index model, showing more information about the importance of index components and improved goodness of fit compared with models with equal weighting. In 3 models, the bayesian index model showed better fit through deviance improvements, while in the other 5 models, the model fit was comparable. Using the Deviance Information Criterion, the bayesian model fit better for 2 screening outcomes (Papanicolaou test, colonoscopy), was equivalent for 5 outcomes, and was an incrementally worse fit for alcohol binge drinking. The second analysis found no effect modification by gender, with none of the interaction terms having exceedance probabilities of 95% or greater. The third analysis showed that individual-level variables did not confound the neighborhood-level deprivation index associations. Among the 5 statistically significant index associations, all persisted when adjusting for individual-level employment, and 4 persisted when adjusting for individual-level income. (Colonoscopy was the exception, where the neighborhood-level deprivation index exceedance probability decreased from 99.9% to 94.1%.) The fourth sensitivity analysis showed no difference to the inference when using the ADI (Table S1); the same cancer control outcomes were statistically significantly associated with both indices and with similar odds ratios. The fifth sensitivity analysis found no difference when considering current smoking status (OR = 1.08, exceedance probability = 98.3%).

## Discussion

This study examined the role of neighborhood-level disadvantage, controlling for individual-level demographic factors, for cancer prevention and control behaviors in a national sample of African American adults. Our models identified statistically significant associations and identified which neighborhood-level factors within the neighborhood-level deprivation index were primary drivers of the relationship expressed in each model. Our expectation that greater levels of neighborhood disadvantage would be associated with a lower likelihood of participants engaging in the cancer control behaviors was supported for alcohol binge drinking, smoking, colonoscopy screening, Papanicolaou testing, and PSA testing.

Greater neighborhood disadvantage was associated with a lower likelihood of ever having had a colonoscopy. This finding is reflected in prior literature, with neighborhood poverty rates and neighborhood disorder being found to have associations with lower colorectal cancer screening uptake.^3^^,^^47^

Among female participants, greater neighborhood disadvantage was statistically significantly associated with a lower likelihood of ever having had a Papanicolaou test, with median household income being the primary neighborhood-level factor driving this relationship. Although a recent analysis of a national dataset found no relationship between neighborhood disadvantage and Papanicolaou test receipt,^7^ other studies have shown neighborhood poverty to be associated with a lower likelihood of Papanicolaou test receipt.^4^ In our study, neighborhood disadvantage was not associated with mammography screening, perhaps because of a ceiling effect. The prominent role of neighborhood-level median household income in screening behaviors could be due to poorer neighborhoods’ limited proximity to screening locations or health-care professionals in less affluent neighborhoods being less likely to recommend colonoscopy to their patients, possible influenced by biases or discrimination.^5^^,^^48^^,^^49^

Among male participants, greater neighborhood disadvantage was statistically significantly associated with a lower likelihood of ever having had a PSA test. A 2020 study also documented this relationship.^9^ The most important neighborhood-level factor in our study was the percentage of residents without some college education, unlike the other cancer screening models, where median household income was key. Although education and income are related, neighborhoods that have some attainment of college education seemed to uniquely influence PSA testing. A more formally educated neighborhood may facilitate information sharing among men, who are generally less connected to the health-care system than women.

Regarding prevention behaviors, greater neighborhood disadvantage was associated with participants being statistically significantly more likely to report multiple recent binge drinking episodes; again, median household income was the primary driver of this relationship. The current literature presents mixed findings on this matter, as evidenced by multiple studies that identify associations between both high and low median household income with heavy drinking.^50-52^ Thus, it is notable that median household income at the neighborhood level played a prominent role in these 2 models of cancer prevention behaviors. In prior literature, median household income was frequently studied as a meaningful predictor of various health-related outcomes, including physical activity,^53^ smoking cessation,^6^ and fruit and vegetable consumption.^54^

Greater neighborhood disadvantage was statistically significantly associated with a higher likelihood of participants having smoked at least 100 cigarettes within their lifetime. Neighborhood percentage of unemployed and percentage of Black residents were the primary drivers of this relationship. Unemployment has previously been linked with increased odds of smoking.^55^^,^^56^ Extant literature has provided evidence for an association between smoking behaviors and factors such as neighborhood social cohesion,^57^ perception of one’s neighborhood,^12^ and neighborhood socioeconomic status.^58^ In addition, smoking and the alcohol outcomes described above may be related to the promotion of these substances through increased density of establishments selling and advertising them.^59^ One recent study conducted in the Southeast found that neighborhood disadvantage was statistically significantly associated with density of tobacco and alcohol retail outlets.^60^ The placement of these outlets has also been found to be more common in neighborhoods with a higher proportion of Black and Hispanic residents.^61^ There are complex relationships between neighborhood disadvantage, density of tobacco and alcohol retail outlets, and systemic racism. Thus, smoking prevention and cessation interventions could benefit from neighborhood-focused approaches and policies to limit access to tobacco products.

Our study has several strengths. First, we evaluated a wide variety of cancer control behaviors, including 4 screening and 4 prevention behaviors, providing a more comprehensive view than studies focusing on fewer outcomes. The prevalence of screening behaviors among participants in our sample reasonably reflected contemporary national rates among African American adults. For certain outcomes (eg, mammogram, Papanicolaou test), observed screening rates were slightly above national averages,^62-64^ but the primary reason for this is that we used ever screening in contrast to past year to provide a clearer distinction between “screeners” and “nonscreeners.” A second strength is our use of the neighborhood-level deprivation index estimated by the bayesian index model, which allows customized estimation of the most important factors in the index for each behavior, enhancing interpretability of neighborhood-level variables. Regarding limitations, the wide geographic scope of the RHIAA study prevented validation of self-reported screening behaviors with medical records, relying instead on self-report. Identified associations may differ in different demographic populations and regions, and findings are not meant to represent all African American adults in the United States. In addition, we cannot rule out residual confounding from unmeasured factors. Finally, we analyzed cancer control outcomes separately. Future research can focus on combined behaviors, recognizing the importance of many such behaviors to minimizing cancer risk.

In conclusion, this study highlights specific neighborhood factors, particularly neighborhood income, associated with various cancer control behaviors in a national sample of African American adults. Undoubtedly, a history of structural inequities, racist policies, and intentional disinvestment has created disparities in neighborhood-level distribution of resources that disproportionately affect this group. Our results underscore the need for targeted, tailored, neighborhood-level interventions addressing specific cancer control behaviors rather than adopting a one-size-fits-all approach.

## Supplementary Material

pkaf015_Supplementary_Data

## Data Availability

Data underlying this article cannot be shared due to ethical/privacy reasons. Summary (eg, Census division-level) and deidentified data may be shared upon request.
