# Supplementary material for: Indices of neighborhood disadvantage and individual cancer control behaviors among African American adults
Source: JNCI Cancer Spectr. 2025 Feb 28;9(1):pkaf015. doi: 10.1093/jncics/pkaf015 (PMC11879093; doi:10.1093/jncics/pkaf015)
Supplement: pkaf015_Supplementary_Data [file pkaf015_supplementary_data.docx]

Supplementary Materials

**Indices of neighborhood disadvantage and individual cancer control behaviors among African American adults**

**Supplementary Methods**

*Outcome measures.* Heavy drinking days were defined as at least five drinks/day for men and four drinks/day for women. MVPA was calculated as the number of minutes of moderate PA plus twice the number of minutes of vigorous PA. Screening questions were sex- and age-specific. For example, only women aged 40 and older were asked if they had had a mammogram.

*Neighborhood-level exposures.* A census tract, having a population averaging 4,000 people and ranging between 1,200-8,000 people and having stable demographic characteristics, is roughly equivalent to a neighborhood established by the Census Bureau^1^. It is smaller than a city but larger than a census block and a city block. Among all the census enumeration units, the census tract is typically merged with large national surveys at the individual level^2–5^.

*Bayesian index model specification.* Specifically, for each outcome variable, we fit the following model: $Y_{i}\sim Bernoulli\left( p_{i} \right)$, and we modeled the log-odds of the probability using the expression:

$$\log\left( \frac{p_{i}}{1-p_{i}} \right)=\beta_{0}+\beta_{1}\sum_{j=1}^{J} w_{j}q_{ij}+\sum_{b=1}^{B} \theta_{b}x_{ib}$$

Here, the cancer control outcome variable $Y_{i}$ takes a value of $1$ if the $i^{th}$ participant has a non-salutary behavior (e.g., having at least three heavy drinking days in the past month, never having had a PSA test, etc.), and $0$ otherwise. The log-odds of the probability of this outcome contain an intercept $\beta_{0}$ and the parameter $\beta_{1}$, which represents the NDI effect. The importance of the $j^{th}$ index component, $j=1, \ldots, J$ is $w_{j}$, and $q_{ij}$ is the $j^{th}$ quantized index component for the $i^{th}$ participant. We quantized components into deciles to account for different scales of measurement, acknowledge uncertainty in estimates, and limit the effects of outliers^6,7^. Fitting these models in the Bayesian paradigm, we assigned prior distributions to parameters as follows. We assigned non-informative Normal priors to the NDI coefficient $\beta_{1}$ as well as all adjustment covariates $\theta_{b}$ that had mean 0 and a standard deviation corresponding to a $95\%$ prior belief that the true odds ratio for the parameter would fall in the interval $\left( \frac{1}{5},5 \right)$. Also, we assigned a flat prior for the intercept $\beta_{0}$. We assigned a $Dirichlet\left( \boldsymbol{1} \right)$ prior to the index weights, such that each weight would be in $\left( 0,1 \right)$ and would sum to $1$. We ran two chains of Markov chain Monte Carlo simulations, burning in a large number of iterations and sampling 5,000 iterations from the posterior distribution for each chain. We assessed convergence with the Gelman-Rubin statistic^8^, considering parameters to have converged if their statistic was $<1.1$.

**References**

1. Census Bureau US. Geographic Terms and Concepts. Accessed August 14, 2019. https://www.census.gov/programs-surveys/geography.html

2. Knott CL, Ghosh D, Williams BR, et al. Do neighborhood characteristics contribute beyond individual demographics to cancer control behaviors among African American adults? *Cancer epidemiology*. 2020;64:101666.

3. Henry KA, Sherman RL, McDonald K, et al. Associations of census-tract poverty with subsite-specific colorectal cancer incidence rates and stage of disease at diagnosis in the United States. *Journal of cancer epidemiology*. 2014;2014.

4. Chang ET, Yang J, Alfaro-Velcamp T, So SK, Glaser SL, Gomez SL. Disparities in liver cancer incidence by nativity, acculturation, and socioeconomic status in California Hispanics and Asians. *Cancer epidemiology, biomarkers & prevention*. 2010;19(12):3106-3118.

5. Freedman VA, Grafova IB, Rogowski J. Neighborhoods and chronic disease onset in later life. *American journal of public health*. 2011;101(1):79-86.

6. Christensen KLY, Carrico CK, Sanyal AJ, Gennings C. Multiple classes of environmental chemicals are associated with liver disease: NHANES 2003--2004. *International journal of hygiene and environmental health*. 2013;216(6):703-709.

7. Hargarten PM, Wheeler DC. Accounting for the uncertainty due to chemicals below the detection limit in mixture analysis. *Environmental research*. 2020;186:109466.

8. Gelman A, Rubin DB. Inference from iterative simulation using multiple sequences. *Statistical science*. 1992;7(4):457-472.

**Supplementary Tables**

**Supplementary Table 1**. Sensitivity analysis using Area Deprivation Index instead of Bayesian Neighborhood Deprivation Index.

| Outcome | Odds Ratio | Exceedance Probability |
| --- | --- | --- |
| Prevention | | |
| **Alcohol** | 1.26 | 99.3 |
| **Smoking** | 1.14 | 100.0 |
| Physical Activity | 1.06 | 85.7 |
| Fruit/Vegetables | 1.05 | 83.8 |
| Screening | | |
| **Colonoscopy** | 1.25 | 99.9 |
| **Pap Smear** | 1.40 | 99.7 |
| **PSA** | 1.24 | 100.0 |
| Mammogram | 1.10 | 76.4 |

Note: Odds ratios correspond to a one-standard deviation increase in ADI values in adjusted logistic regression models using the same covariates as in the main analysis. Outcomes in boldface denote statistically significant associations.

**Supplementary Figures**

**
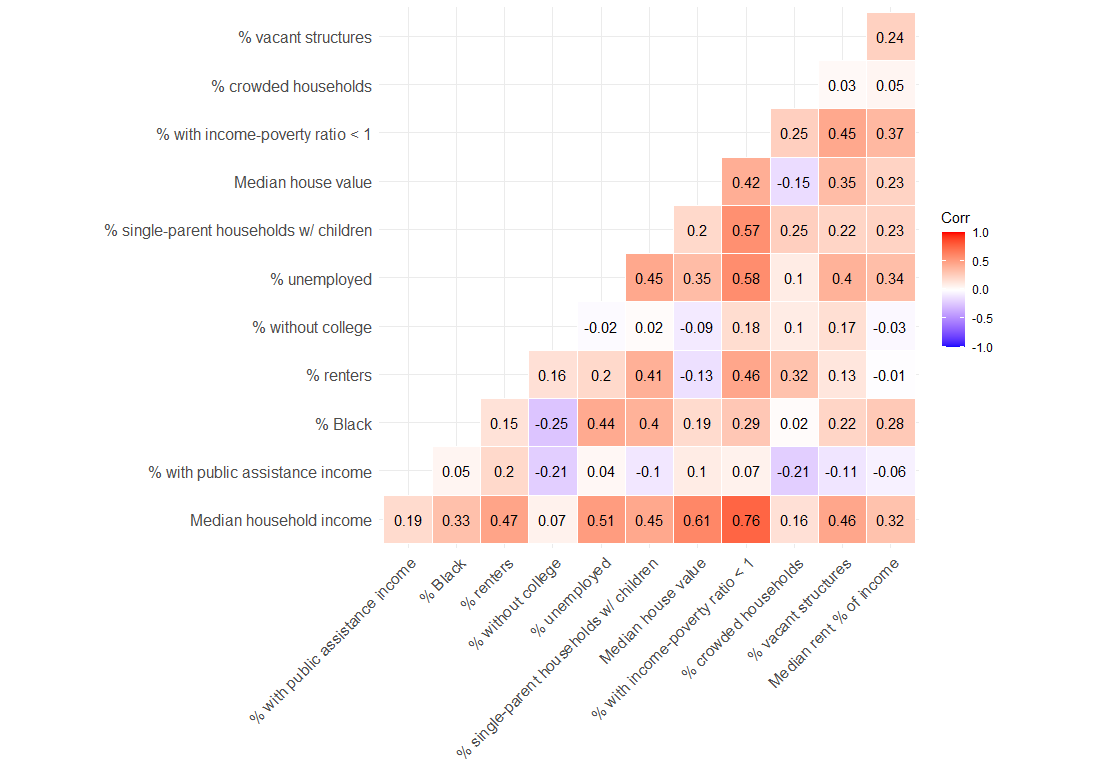
Supplementary Figure 1**. Correlation matrix of components in Bayesian Neighborhood Deprivation Index.

**
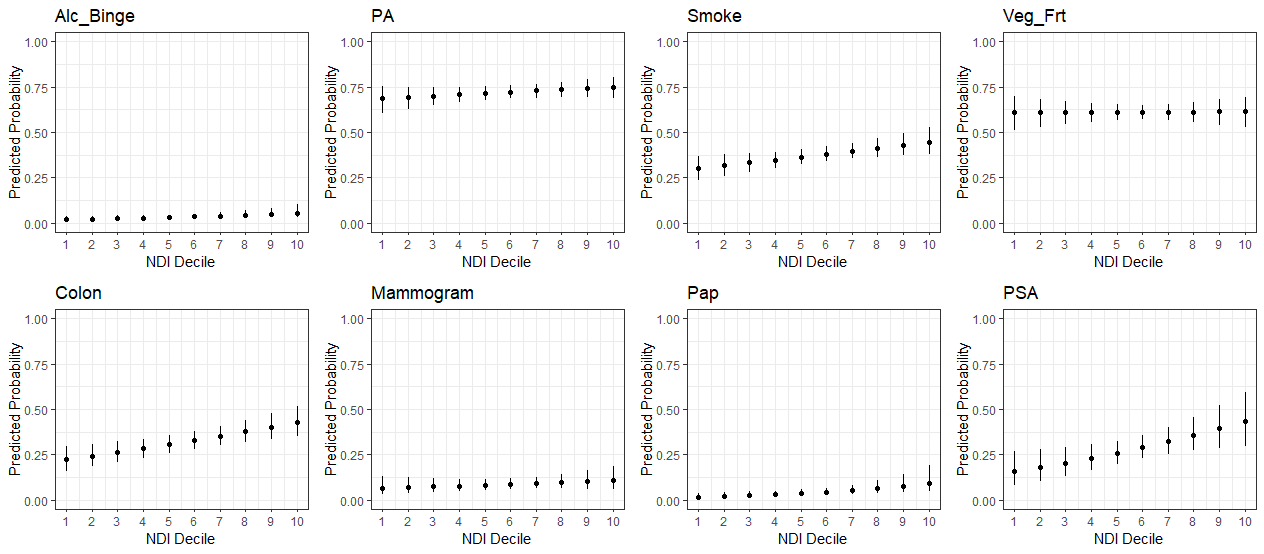
Supplementary Figure 2**. Predicted probabilities of outcomes as a function of NDI deciles.

Note: For each outcome, the predicted probabilities are calculated using the mean age value in the sample, the mode value of education, and the mode value of sex (if applicable). Presented quantities in the figure are the median (dot) and 95% credible interval (line) predicted probability for each decile of the NDI.
